# Cadmium Nephrotoxicity Is Associated with Altered MicroRNA Expression in the Rat Renal Cortex

**DOI:** 10.3390/toxics6010016

**Published:** 2018-03-15

**Authors:** Michael J. Fay, Lauren A. C. Alt, Dominika Ryba, Ribhi Salamah, Ryan Peach, Alexander Papaeliou, Sabina Zawadzka, Andrew Weiss, Nil Patel, Asad Rahman, Zyaria Stubbs-Russell, Peter C. Lamar, Joshua R. Edwards, Walter C. Prozialeck

**Affiliations:** 1 Department of Biomedical Sciences, Midwestern University, 555 31st Street, Downers Grove, IL 60515, USA; lalt@midwestern.edu (L.A.C.A.); dryba24@midwestern.edu (D.R.); rsalamah66@midwestern.edu (R.S.); rpeach38@midwestern.edu (R.P.); apapaeliou97@midwestern.edu (A.P.); szawadzka34@midwestern.edu (S.Z.); aweiss16@midwestern.edu (A.W.); npatel16@midwestern.edu (N.P.); arahman13@midwestern.edu (A.R.); zstubbs-russell11@midwestern.edu (Z.S.-R.); 2 Department of Pharmacology, Midwestern University, 555 31st Street, Downers Grove, IL 60515, USA; plamar@midwestern.edu (P.C.L.); jedwar@midwestern.edu (J.R.E.); wprozi@midwestern.edu (W.C.P.)

**Keywords:** cadmium, microRNAs, nephrotoxicity, biomarkers

## Abstract

Cadmium (Cd) is a nephrotoxic environmental pollutant that causes a generalized dysfunction of the proximal tubule characterized by polyuria and proteinuria. Even though the effects of Cd on the kidney have been well-characterized, the molecular mechanisms underlying these effects have not been fully elucidated. MicroRNAs (miRNAs) are small non-coding RNAs that regulate cellular and physiologic function by modulating gene expression at the post-transcriptional level. The goal of the present study was to determine if Cd affects renal cortex miRNA expression in a well-established animal model of Cd-induced kidney injury. Male Sprague-Dawley rats were treated with subcutaneous injections of either isotonic saline or CdCl_2_ (0.6 mg/kg) 5 days a week for 12 weeks. The 12-week Cd-treatment protocol resulted in kidney injury as determined by the development of polyuria and proteinuria, and a significant increase in the urinary biomarkers Kim-1, β_2_ microglobulin and cystatin C. Total RNA was isolated from the renal cortex of the saline control and Cd treated animals, and differentially expressed miRNAs were identified using µParaflo^TM^ microRNA microarray analysis. The microarray results demonstrated that the expression of 44 miRNAs were significantly increased and 54 miRNAs were significantly decreased in the Cd treatment group versus the saline control (*t*-test, *p* ≤ 0.05, *N* = 6 per group). miR-21-5p, miR-34a-5p, miR-146b-5p, miR-149-3p, miR-224-5p, miR-451-5p, miR-1949, miR-3084a-3p, and miR-3084c-3p demonstrated more abundant expression and a significant two-fold or greater increased expression in the Cd-treatment group versus the saline control group. miR-193b-3p, miR-455-3p, and miR-342-3p demonstrated more abundant expression and a significant two-fold or greater decreased expression in the Cd-treatment group versus the saline control group. Real-time PCR validation demonstrated (1) a significant (*t*-test, *p* ≤ 0.05, *N* = 6 per group) increase in expression in the Cd-treated group for miR-21-5p (2.7-fold), miR-34a-5p (10.8-fold), miR-146b-5p (2-fold), miR-224-5p (10.2-fold), miR-3084a-3p (2.4-fold), and miR-3084c-3p (3.3-fold) and (2) a significant (*t*-test, *p* ≤ 0.05, *N* = 6 per group) 52% decrease in miR-455-3p expression in the Cd-treatment group. These findings demonstrate that Cd significantly alters the miRNA expression profile in the renal cortex and raises the possibility that dysregulated miRNA expression may play a role in the pathophysiology of Cd-induced kidney injury. In addition, these findings raise the possibility that Cd-dysregulated miRNAs might be used as urinary biomarkers of Cd exposure or Cd-induced kidney injury.

## 1. Introduction

The nephrotoxic heavy metal cadmium (Cd) is a Group 1 carcinogen and currently ranked 7th on the 2017 Agency for Toxic Substances and Disease Registry (ATSDR) and EPA list of hazardous substances [1]. Industrial activities have resulted in increases in the concentrations of Cd in the environment. Human exposure can occur by inhalation in the workplace, the ingestion of contaminated food and water, and smoking tobacco [2]. Circulating Cd that is bound to low-molecular-weight proteins or thiol compounds is filtered at the glomerulus and taken up by proximal tubule epithelial cells, and chronic low-level human exposure to Cd results in proximal tubule accumulation [3]. When a critical Cd threshold of 150–200 µg/g wet weight (equivalent to 450–600 µg/g dry weight) is reached, toxic injury can occur, which is manifested by a generalized reabsorptive dysfunction resulting in polyuria and low-molecular-weight proteinuria [4,5].

While the toxic effects of Cd on the proximal tubule are well documented, the molecular mechanisms associated with Cd-induced kidney injury have not been fully elucidated. Prior to causing cell death, Cd has been shown to induce oxidative stress, promote cytoskeletal reorganization, disrupt cadherin-dependent cell–cell adhesion, decrease transepithelial electrical resistance, activate various cellular signaling pathways, and induce endoplasmic reticulum stress and autophagy in proximal tubule epithelial cells [6,7]. Even though Cd can affect a wide variety of cellular processes, it is not well established how novel regulators such as microRNAs (miRNAs) might be involved in Cd-induced proximal tubule epithelial cellular injury.

MicroRNAs are evolutionarily conserved small (20–25 nt) non-protein coding RNAs that inhibit gene expression at the post-transcriptional level by blocking mRNA translation or promoting mRNA degradation within a cell [8,9]. These non-protein coding miRNAs can have a major impact on cellular function, as a single miRNA can interact with hundreds of different protein-coding mRNAs, and a single protein-coding mRNA can be affected by multiple miRNAs [10,11]. MicroRNAs are known to be involved in kidney development, kidney homeostasis, and the pathophysiology of kidney disease [12,13,14,15,16]. There is also evidence that dysregulated miRNA expression is associated with kidney injury in both rodent and human studies, and that miRNAs have the potential to serve as urinary biomarkers of kidney injury [14,16,17,18,19,20]. However, there is a lack of information concerning the role of miRNAs in Cd-induced nephrotoxicity. The purpose of this research study was to use a well-established sub-chronic animal model to determine if Cd-induced nephrotoxicity is associated with dysregulated miRNA expression in the renal cortex [21].

## 2. Material and Methods

### 2.1. Animal Protocol

The animal protocol used for these studies is a well-established and well-characterized sub-chronic treatment protocol for producing Cd-induced nephrotoxicity in the rat [21]. The animal research was conducted in compliance with the United States NIH Guide for the Care and Use of Laboratory Animals (National Research Council of the National Academies, 2011), and all studies were approved by the Institutional Animal Care and Use Committee of Midwestern University. Adult male Sprague-Dawley rats weighing 250–300 g were purchased from Envigo (Indianapolis, IN, USA). The rats were housed socially with two rats per plastic cage, and the animals were maintained on a 12/12 h light/dark cycle. For the Cd treatment group, animals (*N* = 6) received daily (Monday–Friday) subcutaneous injections of CdCl_2_ at a Cd dose of 0.6 mg (5.36 µmoles)/kg in 0.25–0.35 mL isotonic saline for 12 weeks, while the vehicle control animals (*N* = 6) received daily injections of isotonic saline. At the end of the 12-week treatment protocol, animals were placed in individual metabolic cages for 24 h to collect urine samples. Animals were allowed free access to water and food ad libitum, with the exception that food was restricted when the animals were in the metabolic cages. Before the start of the dosing regimen, the Cd concentration in the stock solution was confirmed by Chemical Solutions, Inc. (Harrisburg, PA, USA) using the technique of inductively coupled plasma mass spectrometry as previously described [22]. At the end of the protocol, the animals were anesthetized with ketamine/xylazine (67/7 mg/kg) by intraperitoneal injection and euthanized by exsanguination and pneumothorax while under anesthesia. Prior to exsanguination and pneumothorax, the kidneys were removed and processed for RNA isolation as described below. 

### 2.2. Biomarker Determination

The 24 h urine was collected and portioned into 0.5–1.0 mL aliquots. The aliquots were frozen at −80 °C and later assayed for protein, creatinine, and the biomarkers of interest. In some cases, prior to freezing, the urine aliquots were stabilized in proprietary buffers and other reagents that are recommended for MAGPIX-based assays that were used for some of the analyses. The urinary levels of cystatin C, Kim-1, and β_2_ microglobulin were determined by microsphere-based Luminex xMAP technology using the MagPix xPONENT 4.1 equipment (Luminex Corp., Austin, TX, USA) The Multiplex technology allows for the determination of multiple analytes in a single sample and provides much greater sensitivities and dynamic ranges than commonly used ELISAs. This technique is similar to the assay that has been used to determine urinary levels of Kim-1 in our previous studies [21,23,24]. Urinary levels of creatinine were determined using a previously described colorimetric method [25]. Urinary protein levels were determined using the Bradford Coomassie blue assay as previously described (Thermo Fisher Scientific, Waltham, MA, USA) [21]. With the dosing protocol used in these studies, Cd-treated animals tended to gain less weight than control animals [21]. Accordingly, all urinary parameters were expressed as units excreted per kg body weight per 24 h.

### 2.3. RNA Isolation

Renal cortices were removed and snap frozen in liquid nitrogen. Frozen tissues were placed in pre-chilled (−80 °C) RNA*later*^®^-ICE Frozen Tissue Transition Solution (Invitrogen by Thermo Fisher Scientific) and stored at −80 °C until samples were processed for RNA isolation. Total RNA was isolated from the tissues using the mirVana™ miRNA Isolation Kit (Invitrogen by Thermo Fisher Scientific) following the manufacturer’s recommended protocol. The integrity of the RNA samples was evaluated using a Thermo Scientific™ NanoDrop™ 2000 spectrophotometer, (Thermo Fisher Scientific) and by examining 28S and 18S ribosomal RNA bands using denaturing agarose gel electrophoresis and ethidium bromide staining.

### 2.4. µParaflo^TM^ MicroRNA Microarray Assay

Microarray analysis for rat miRNAs (Sanger miRBase release 21) was performed using µParaflo™ microfluidic chip technology (LC Sciences, Houston, TX, USA). Total RNA (1 µg) from Cd-treated and saline control rats (*N* = 6 per group) was 3′-extended with a poly(A) tail, and an oligonucleotide was then ligated to the poly(A) tail for subsequent fluorescent dye staining. Hybridization was performed overnight on a µParaflo™ microfluidic chip using a microcirculation pump (Atactic Technologies, Houston, TX, USA) [26,27]. The microfluidic chip contained 761 unique detection probes made by in situ synthesis using Photogenerated Reagent (PGR) chemistry, and the probes consisted of a chemically modified nucleotide segment complementary to a target miRNA from miRbase (http://mirbase.org) or control RNA with a polyethylene glycol spacer segment. The hybridization melting temperatures were balanced by chemical modifications of the detection probes. Hybridization was performed using 100 µL of 6x SSPE buffer (0.90 M NaCl, 60 mM Na_2_HPO_4_, 6 mM EDTA, pH 6.8) containing 25% formamide at 40 °C. After RNA hybridization, tag-conjugating Alexa Fluor^®^ 647 dye was circulated through the microfluidic chip for dye staining. Fluorescence images were collected using a laser scanner (GenePix 4000B, Molecular Devices, San Jose, CA, USA) and digitized using Array-Pro image analysis software (Version 4.0, Media Cybernetics, Rockville, MD, USA, 1981). Data were analyzed by first subtracting the background and then normalizing the signals using a LOWESS filter (locally weighted regression) [28]. Statistical analysis was performed by LC Sciences using an unpaired *t* test (*N* = 6 per group, *p* ≤ 0.05).

### 2.5. MicroRNA Real-Time PCR

The cDNA template for PCR was prepared using 10 ng of total RNA sample and the TaqMan^®^ Advanced miRNA cDNA Synthesis kit (Thermo Fisher Scientific) following the manufacturer’s recommended protocol. MicroRNA expression in the samples was assessed using TaqMan^®^ Advanced miRNA assays and an Applied Biosystems QuantStudio 5 real-time PCR system. Selected miRNAs that demonstrated a statistically significant (*p* ≤ 0.05) altered expression using µParaflo™ microRNA microarray were validated using the following TaqMan^®^ Advanced miRNA assays: miR-21-5p (rno481342_mir), miR-34a-5p (rno481304_mir), miR-146b-5p (rno480941_mir), miR-224-5p (rno481010_mir), miR-3084a-3p (rno481040_mir), miR-3084c-3p (rno481313_mir), and miR-455-3p (rno481396_mir). As an endogenous control, miR-26a-5p (hsa477995_mir) was used since this miRNA was abundantly expressed in the rat renal cortex and the expression levels were not affected with Cd treatment. As an additional validation of the µParaflo™ microRNA microarray assay, the expression of miR-423-5p (rno481159_mir) was examined by real-time PCR since the expression of this miRNA was not significantly altered with Cd treatment. The fold change in miRNA expression between saline control and Cd-treated samples was determined using the comparative CT method as previously described using QuantStudio™ Design Analysis Software (Version 1.4, Applied Biosystems by Thermo Fisher Scientific, Carlsbad, CA, USA, 2016) [29,30]. Statistical analysis was performed on the 2^−ΔCT^ values using an unpaired *t*-test (*N* = 6 per group, *p* ≤ 0.05) with GraphPad Prism software (Version 7.00, GraphPad Software Inc., La Jolla, CA, USA, 2016). 

## 3. Results

### 3.1. Cd-Induced Kidney Injury in a Sub-Chronic Rat Model

**Figure 1:**
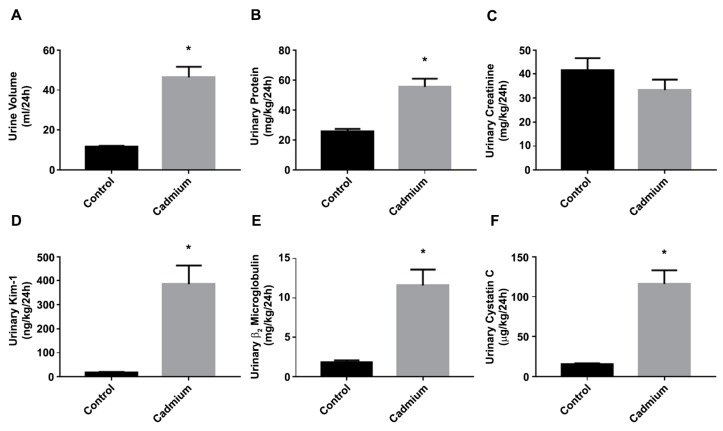
Assessment of Cd-induced nephrotoxicity in a 12-week sub-chronic rat model. Male Sprague-Dawley rats received daily subcutaneous injections of Cd (0.6 mg/kg/day) 5-days a week for 12 weeks, while the controls received injections of isotonic saline. (**A**) Urine volume; (**B**) urinary protein; (**C**) urinary creatinine; (**D**) urinary Kim-1; (**E**) urinary β_2_ microglobulin; (**F**) urinary cystatin C. The data are mean ± SEM; an asterisk (*) indicates statistical significance from the saline control (*N* = 6 per group, unpaired *t*-test, *p* ≤ 0.05).

The 12-week sub-chronic Cd-treatment protocol resulted in kidney injury. In the Cd-treated animals, there was a significant 4-fold increase in the 24 h urine volume (Figure 1A) and a significant 2.2-fold increase in the 24 h urinary protein excretion (Figure 1B), with no significant change in the urinary creatinine excretion (Figure 1C). There was also a significant 21.7-fold increase in urinary Kim-1 (Figure 1D), a significant 6.1-fold increase in urinary β_2_ microglobulin (Figure 1E), and a significant 7.4-fold increase in urinary cystatin C (Figure 1F). These changes in urinary parameters and biomarkers were essentially identical to reported recent studies from the Prozialeck laboratory [21,23,24,31].

### 3.2. µParaflo™ MicroRNA Microarray

**Figure 2:**
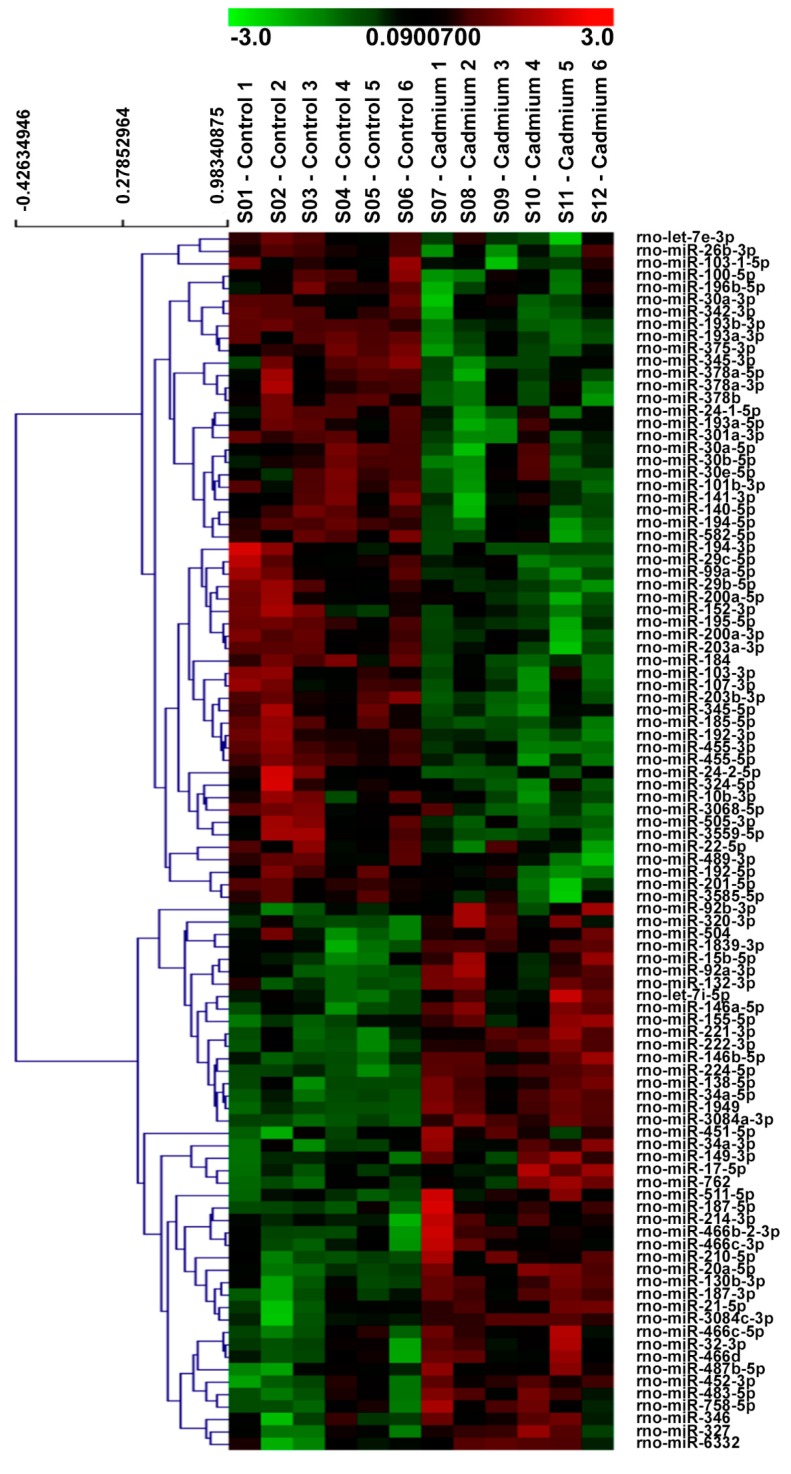
Effects of Cd on miRNA expression in the rat renal cortex. Microarray heat map demonstrating significant differences in the expression of miRNAs in the renal cortex of Cd-treated (0.6 mg/kg/day, 5 days per week for 12 weeks) male Sprague-Dawley rats versus saline controls. Cadmium significantly decreased the expression of 54 miRNAs and increased the expression of 44 miRNAs (*N* = 6 per group, unpaired *t*-test, *p* ≤ 0.05).

To determine if Cd alters the miRNA profile in the renal cortex of 12-week Cd-treated rats, the miRNA expression profile between Cd-treated and saline control rats was compared using µParaflo™ microRNA microarray analysis. As shown in the heat map in Figure 2, the expression of 54 miRNAs were significantly decreased in the Cd-treated group versus the saline control group, while the expression of 44 miRNAs were significantly increased. More detailed information concerning the miRNAs demonstrating significantly increased expression or decreased expression is shown in Table 1 and Table 2, respectively. miR-21-5p, miR-34a-5p, miR-146b-5p, miR-149-3p, miR-224-5p, miR-451-5p, miR-1949, miR-3084a-3p, and miR-3084c-3p demonstrated more abundant expression and a two-fold or greater significant increase in expression with Cd treatment. miR-193b-3p, miR-455-3p, and miR-342-3p demonstrated more abundant expression and a two-fold or greater significant decreased expression with Cd treatment.

### 3.3. Real-Time PCR Validation

**Figure 3:**
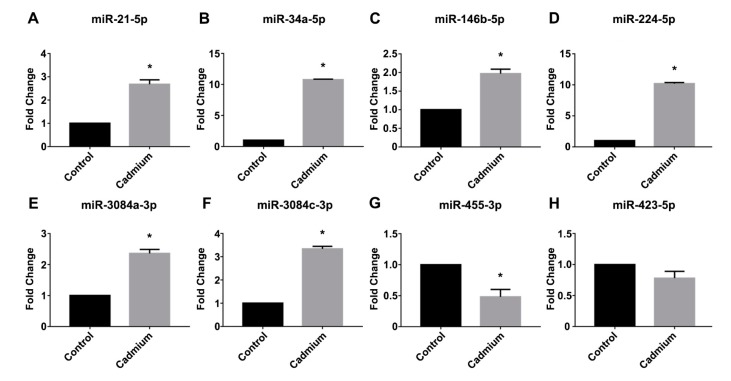
Real-time PCR validation of Cd-dysregulated miRNAs. TaqMan^®^ Advanced miRNA assays were used to validate selected Cd-dysregulated miRNAs. (**A**) miR-21-5p; (**B**) miR-34a-5p; (**C**) miR-146b-5p; (**D**) miR-224-5p; (**E**) miR-3084a-3p; (**F**) miR-3084c-3p; (**G**) miR-455-3p; (**H**) miR-423-5p. The comparative CT method was used to determine the fold change (±SEM), and an asterisk (*) indicates a statistically significant change in expression in the Cd-treated group versus the saline control (*N* = 6 per group, unpaired *t*-test, *p* ≤ 0.05).

Real-time PCR was used to validate the expression of selected miRNAs that demonstrated more abundant expression and demonstrated a two-fold or greater change in expression as determined by the µParaflo™ microRNA microarray analysis. As shown in Figure 3, real-time PCR demonstrated a significant increase in the Cd-treated group for the following miRNAs: a 2.7-fold increase in miR-21-5p (Figure 3A), a 10.8-fold increase in miR-34a-5p (Figure 3B), a 2-fold increase in miR-146b-5p (Figure 3C), a 10.2-fold increase in miR-224-5p (Figure 3D), a 2.4-fold increase in miR-3084a-3p (Figure 3E), and a 3.3-fold increase in miR-3084c-3p (Figure 3F). By contrast, real-time PCR validation demonstrated a significant 52% decrease in miR-455-3p expression in the Cd-treatment group (Figure 3G). As a control, we also examined the expression of a miRNA (miR-423-5p) that did not demonstrate altered expression in the Cd-treatment group versus the saline control group according to the microarray analysis, and real-time PCR analysis confirmed there was no significant difference in the expression of miR-423-5p between the saline control and Cd-treatment group (Figure 3H).

## 4. Discussion

The purpose of this research was to determine if Cd-induced nephrotoxicity is associated with dysregulated miRNA expression in the renal cortex. As a research model of Cd-induced nephrotoxicity, we used a well-characterized sub-chronic in vivo research model in which male Sprague-Dawley rats received daily subcutaneous injections of CdCl_2_ (0.6 mg/kg/day, 5 days per week for 12 weeks) [21]. The Cd-induced damage to the proximal tubule was confirmed by demonstrating polyuria and proteinuria in the Cd-treatment group versus the saline control group [21]. The fact that the 12-week Cd-treated animals developed polyuria and proteinuria without a significant change in creatinine excretion supports the fact that the Cd-induced kidney injury is at the level of the proximal tubule [5,6,21,24,31,32]. Some of the urinary biomarkers that have been used to monitor kidney injury include Kim-1, β_2_ microglobulin, and cystatin C [21,31,33,34,35,36]. All three of these urinary biomarkers have previously been shown to be elevated in the animal model of Cd-induced kidney injury used for this study, and consistent with these previous findings, all three biomarkers were significantly increased in our study [21,31].

The microarray results demonstrated a significant increase in the expression of 44 miRNAs in the renal cortices from the Cd-treated animals versus the saline control group. miR-21-5p, miR-34a-5p, miR-146b-5p, miR-149-3p, miR-224-5p, miR-451-5p, miR-1949, miR-3084a-3p, and miR-3084c-3p were more abundantly expressed and demonstrated a two-fold or greater increased expression in the Cd-treatment group. We used real-time PCR to confirm the increased expression of miR-21-5p, miR-34a-5p, mir-146b-5p, miR-224-5p, miR-3084a-3p, and miR-3084c-3p in the renal cortices from Cd-treated animals versus the saline controls. The elevated level of some of these miRNAs have also been demonstrated in other models of kidney injury. Previous research demonstrated increased expression of miR-21 in the kidneys of mice with ischemia/reperfusion injury or gentamicin-induced kidney injury, and miR-21 levels were also increased in the urine of patients with acute kidney injury versus healthy patients [37]. The cellular effects of elevated miR-21 on proximal tubule epithelial cells may be both protective and/or damaging as miR-21 has been shown to limit damage resulting from reactive oxygen species and affect apoptosis and fibrosis [20,38,39]. Several rodent studies have demonstrated that drug-induced kidney injury is associated with increased expression of p53-responsive miR-34a, and this miRNA has been shown to suppress autophagy in tubular epithelial cells in a mouse model of ischemia/reperfusion-induced acute kidney injury [40,41,42,43]. The expression of miR-146-5p was shown to be increased with fibrosis in a mouse model of folic-acid-induced kidney injury, and in mouse models of ischemia/reperfusion injury and unilateral urethral obstruction-induced fibrosis [44]. Additionally, miR-146-5p expression was increased in human renal cortices with documented severe kidney injury or fibrosis [44]. Although not directly linked to kidney injury, upregulation of miR-149-3p may decrease clonogenicity and induce apoptosis by targeting polo-like kinase 1 (PLK1) [45]. Additionally, miR-149-3p inhibits the proliferation, migration, and invasion of bladder cancer cells by targeting the S100A4 protein, which is involved with cellular differentiation, motility, and regulating transcription [46]. Upregulation of miR-224-5p has been found to occur as part of the protective adaptive response of hepatocytes during acetaminophen-induced toxicity, and this upregulation of miR-224-5p was associated with suppression of drug metabolizing enzyme levels [47]. Using a streptozotocin-induced diabetic rat model, Mohan et al. demonstrated the utility of urinary exosomal miR-451-5p as an early biomarker of diabetes-associated nephropathy, and the elevated levels of miR-451-5p appeared to be protective against kidney fibrosis [48]. Increased expression of miR-1949 was previously shown in injured kidney tissue in a rat model of deep hypothermic circulatory arrest [49]. Previous research demonstrated increased expression of miR-3084-3p in the renal cortices of mice treated with ^177^Lu-octreotate, and this radionuclide therapy used for treating neuroendocrine tumors is known to cause renal toxicity at the level of the proximal tubule [50,51].

Although it did not meet the criteria of two-fold or greater increased expression, miR-320-3p demonstrated significantly increased expression in the Cd-treatment group. Previous research has identified β-catenin mRNA as a target of miR-320 [52]. This is relevant because our research group demonstrated using both in vitro and in vivo research models that prior to inducing cell death, Cd disrupts cadherin-dependent cell–cell adhesions with a resulting loss of cadherin and β-catenin at cell–cell contacts [6,53,54,55,56,57].

The microarray results demonstrated significantly decreased expression of 54 miRNAs in the renal cortices from the Cd-treated group versus the saline control group, and miR-193b-3p, miR-455-3p, and miR-342-3p demonstrated a more abundant expression level and a two-fold or greater decreased expression in the Cd-treatment group versus the saline control group. As shown here, the decreased expression of miR-455-3p in the renal cortices from Cd-treatment animals was confirmed by real-time PCR. Fabbri et al. treated HepG2 human hepatoma cells with 10 µM Cd for 24 h and reported decreased expression of 12 miRNAs, including members of the let-7 family (let-7a, let-7b, let-7e, and let-7g) and miR-455-3p [58]. The microarray results presented here also demonstrated decreased expression of let-7e-3p, and both the microarray and real-time PCR validation from our study demonstrated decreased expression of miR-455-3p. The top pathways that were identified to be affected by the altered miRNA expression profile in the Cd-treated HepG2 cells were focal adhesion and the MAPK signaling pathway, and members of the let-7 miRNA family are known to serve a tumor suppressor role [58,59]. Another study used lentiviruses to silence β-catenin in gastric cancer cell lines and found the dysregulated expression of a number of miRNAs including increased expression of miR-210 and decreased expression of miR-455-3p [60]. Both miR-210-5p and miR-455-3p were also dysregulated in our study, and we have previously shown that Cd alters β-catenin sub-cellular localization and activity in NRK-52E rat proximal tubule epithelial cells and causes a loss of β-catenin at cell–cell contacts [6,53,54,55,56,57]. miRNA-193b-3p was found to be downregulated in the renal cortices from Cd-treated animals, and downregulation of this miRNA has been shown to promote autophagy and cell survival in mouse motor neuron-like cells [61]. Additionally, downregulated miR-342-3p expression is part of a shared dysregulated miRNA expression profile in both mutated non-invasive follicular thyroid neoplasms with papillary-like nuclear features (NIFTPs) and infiltrative and invasive follicular variants of papillary thyroid carcinomas (FVPTCs); and in contrast to wild type NIFTPs, this dysregulated miRNA expression profile is predicted to promote an invasive-like phenotype by altering cell adhesion and cell migration pathways [62].

It is important to note that the sub-chronic animal model of Cd-induced kidney injury that was employed for the present studies is similar to protocols that have been used by other investigators [24]. With this sub-chronic Cd-treatment protocol, the classic symptoms of polyuria and proteinuria without a change in creatinine excretion appear after 9–10 weeks of the Cd-dosing protocol. Even after 12 weeks, the level of injury in the proximal tubule is relatively mild; as a result, this animal model is useful for identifying cellular alterations and biomarkers at early stages of Cd-induced proximal tubule injury prior to overt dysfunction. The fact that changes in miRNA expression are quite pronounced at 12 weeks, when the level of injury is mild, suggests that the changes in miRNA expression represent early events in the pathophysiology of Cd-induced kidney injury. More detailed time course studies, beyond the scope of this report, will be needed to clarify this issue. An important consideration for this research is the tissue source of the Cd-dysregulated miRNAs, as the renal cortices were obtained from non-perfused animals. In our experience with this animal model, there is limited blood associated with the isolated renal cortices compared to the amount of renal tissue. However, to confirm the renal source of the miRNAs, future studies will be performed using in situ hybridization.

## 5. Conclusions

The results of these studies demonstrate that Cd-induced nephrotoxicity is associated with dysregulated miRNA expression in the rat renal cortex. These dysregulated miRNAs may serve a protective role and/or promote injury of the Cd-exposed proximal tubule epithelial cells. Identifying the mRNA targets of these dysregulated miRNAs and the associated cellular signaling pathways may help to identify novel therapeutic strategies for preventing and treating kidney disease. In addition to blocking gene expression at the post-transcriptional level, these Cd-dysregulated miRNAs may also serve as useful non-invasive urinary biomarkers of Cd exposure or Cd-induced kidney injury. Future studies from our research group will determine if the expression of these miRNAs are dysregulated in the renal cortex prior to the Cd-induced kidney injury that is seen after 12 weeks of Cd treatment (0.6 mg/kg/day, 5 days per week), and can be used as non-invasive urinary biomarkers of Cd-induced kidney injury. Finally, we will utilize bioinformatics and an in vitro research model to determine the mRNA targets and cellular effects of these dysregulated miRNAs through the use of both miRNA mimics and inhibitors. These studies will provide useful information regarding the molecular mechanisms of Cd-induced damage to proximal tubule epithelial cells, and will determine if miRNAs can be used as early non-invasive biomarkers of Cd-induced damage to the proximal tubule.

## Figures and Tables

**Table 1:** MicroRNAs with significantly increased expression in the renal cortex of Cd-treated rats as determined by µParaflo™ microRNA microarray analysis.

MicroRNA	*p*-Value	Control Mean (RFS *)	Cadmium Mean (RFS *)	Log 2 (Cadmium/Control)
miR-3084a-3p	1.05 × 10^−6^	1019	3016	1.57
miR-34a-5p	4.57 × 10^−6^	99	612	2.62
miR-1949	1.10 × 10^−5^	41	326	2.98
miR-224-5p	3.75 × 10^−5^	12	390	5.06
miR-222-3p	3.00 × 10^−4^	622	1127	0.86
miR-221-3p	3.95 × 10^−4^	968	1643	0.76
miR-146b-5p	8.79 × 10^−4^	200	558	1.48
miR-210-5p	1.81 × 10^−3^	1140	1740	0.61
miR-20a-5p	1.87 × 10^−3^	1179	1756	0.58
miR-146a-5p	2.89 × 10^−3^	3840	5884	0.62
miR-3084c-3p	4.34 × 10^−3^	1174	3419	1.54
miR-92a-3p	6.52 × 10^−3^	1083	1926	0.83
miR-21-5p	6.98 × 10^−3^	10,943	22,388	1.03
miR-466b-2-3p	7.25 × 10^−3^	2101	3143	0.58
miR-320-3p	1.18 × 10^−2^	1377	1882	0.45
miR-15b-5p	1.29 × 10^−2^	1032	1647	0.67
miR-466c-3p	1.29 × 10^−2^	3427	5220	0.61
miR-214-3p	1.64 × 10^−2^	1582	2094	0.40
miR-483-5p	1.74 × 10^−2^	711	1184	0.74
miR-149-3p	1.78 × 10^−2^	1573	3796	1.27
let-7i-5p	2.67 × 10^−2^	3498	4619	0.40
miR-762	2.84 × 10^−2^	915	1702	0.90
miR-466d	3.47 × 10^−2^	370	675	0.87
miR-346	3.57 × 10^−2^	315	440	0.48
miR-17-5p	3.60 × 10^−2^	877	1269	0.53
miR-451-5p	3.63 × 10^−2^	552	1177	1.09
miR-92b-3p	3.81 × 10^−2^	471	759	0.69
miR-466c-5p	3.83 × 10^−2^	229	389	0.76
miR-32-3p	4.07 × 10^−2^	622	1144	0.88
Statistically significant transcripts with low signals (signal < 500)				
miR-138-5p	4.00 × 10^−4^	43	140	1.71
miR-130b-3p	7.36 × 10^−4^	12	59	2.25
miR-187-3p	3.82 × 10^−3^	84	242	1.53
miR-155-5p	6.57 × 10^−3^	33	197	2.57
miR-1839-3p	7.09 × 10^−3^	293	417	0.51
miR-187-5p	8.23 × 10^−3^	66	114	0.79
miR-132-3p	9.57 × 10^−3^	59	189	1.66
miR-34a-3p	1.08 × 10^−2^	7	24	1.86
miR-452-3p	1.71 × 10^−2^	5	27	2.36
miR-511-5p	1.99 × 10^−2^	36	90	1.32
miR-758-5p	2.08 × 10^−2^	203	281	0.47
miR-487b-5p	2.13 × 10^−2^	29	69	1.22
miR-327	2.19 × 10^−2^	28	51	0.84
miR-504	4.10 × 10^−2^	98	136	0.47
miR-6332	4.30 × 10^−2^	16	28	0.82

* Relative fluorescent signal.

**Table 2:** MicroRNAs with significantly decreased expression in the renal cortex of Cd-treated rats as determined by µParaflo™ microRNA microarray analysis.

MicroRNA	*p*-Value	Control Mean (RFS *)	Cadmium Mean (RFS *)	Log 2 (Cadmium/Control)
miR-193b-3p	2.29 × 10^−5^	445	137	−1.70
miR-185-5p	2.81 × 10^−5^	1150	628	−0.87
miR-455-3p	2.06 × 10^−4^	764	258	−1.57
miR-195-5p	4.76 × 10^−4^	4374	3035	−0.53
miR-200a-3p	2.31 × 10^−3^	5998	3725	−0.69
miR-101b-3p	2.56 × 10^−3^	465	285	−0.71
miR-194-5p	2.72 × 10^−3^	13,390	7697	−0.80
miR-99a-5p	2.79 × 10^−3^	5468	3596	−0.60
miR-505-3p	3.59 × 10^−3^	539	371	−0.54
miR-342-3p	4.25 × 10^−3^	1871	845	−1.15
miR-203a-3p	5.21 × 10^−3^	1327	730	−0.86
miR-378a-3p	6.43 × 10^−3^	2576	1616	−0.67
miR-378a-5p	6.67 × 10^−3^	416	233	−0.83
miR-140-5p	7.56 × 10^−3^	403	228	−0.82
miR-378b	9.43 × 10^−3^	1985	1298	−0.61
miR-103-3p	1.73 × 10^−2^	2717	2000	−0.44
miR-107-3p	1.74 × 10^−2^	2781	2052	−0.44
miR-192-5p	2.31 × 10^−2^	13,962	11,183	−0.32
miR-152-3p	2.98 × 10^−2^	971	683	−0.51
miR-100-5p	3.39 × 10^−2^	2133	1318	−0.70
miR-30a-3p	3.73 × 10^−2^	837	552	−0.60
miR-30a-5p	3.81 × 10^−2^	15,805	12,197	−0.37
miR-22-5p	3.84 × 10^−2^	939	812	−0.21
miR-30b-5p	3.93 × 10^−2^	14,704	11,704	−0.33
miR-196b-5p	4.03 × 10^−2^	464	318	−0.54
miR-489-3p	4.21 × 10^−2^	485	311	−0.64
miR-30e-5p	4.68 × 10^−2^	10,074	6429	−0.65
Statistically significant transcripts with low signals (signal < 500)				
miR-203b-3p	6.03 × 10^−5^	146	31	−2.25
miR-192-3p	7.66 × 10^−5^	299	105	−1.50
miR-193a-3p	2.05 × 10^−4^	328	104	−1.65
miR-455-5p	3.55 × 10^−4^	70	17	−2.05
miR-184	6.06 × 10^−4^	27	5	−2.52
miR-375-3p	7.44 × 10^−4^	39	11	−1.86
miR-345-5p	1.04 × 10^−3^	183	103	−0.82
miR-29b-5p	2.03 × 10^−3^	148	78	−0.92
miR-301a-3p	3.09 × 10^−3^	122	60	−1.03
miR-3559-5p	5.48 × 10^−3^	298	161	−0.89
miR-582-5p	9.16 × 10^−3^	165	99	−0.73
miR-345-3p	9.25 × 10^−3^	58	36	−0.70
miR-24-1-5p	1.07 × 10^−2^	98	53	−0.88
miR-29c-5p	1.07 × 10^−2^	274	161	−0.77
miR-24-2-5p	1.12 × 10^−2^	276	181	−0.61
miR-10b-3p	1.54 × 10^−2^	200	121	−0.72
miR-3068-5p	1.86 × 10^−2^	162	113	−0.52
miR-200a-5p	1.87 × 10^−2^	133	73	−0.86
miR-201-5p	2.26 × 10^−2^	67	33	−1.00
miR-141-3p	2.41 × 10^−2^	171	83	−1.05
miR-194-3p	2.63 × 10^−2^	83	44	−0.92
miR-324-5p	2.73 × 10^−2^	243	180	−0.43
miR-26b-3p	3.38 × 10^−2^	27	10	−1.47
miR-193a-5p	3.45 × 10^−2^	20	5	−2.12
miR-3585-5p	3.50 × 10^−2^	67	34	−0.98
let-7e-3p	4.06 × 10^−2^	47	23	−1.00
miR-103-1-5p	4.71 × 10^−2^	32	22	−0.52

* Relative fluorescent signal.
